# Quantum phase transitions: from ordered magnets to spin glasses

**DOI:** 10.1093/nsr/nwag038

**Published:** 2026-01-19

**Authors:** Michael Smidman, Huiqiu Yuan

**Affiliations:** Center for Correlated Matter and School of Physics, Zhejiang University, China; Center for Correlated Matter and School of Physics, Zhejiang University, China

The properties of classical phase transitions that occur at finite temperatures are governed by thermal fluctuations, leading to a host of universal behaviors that are independent of the underlying microscopic physics. In some strongly correlated electron systems, in which there are significant many-body electron interactions, it is possible to apply a non-thermal parameter to continuously suppress a thermodynamic phase transition to zero-temperature at a quantum-critical point (QCP). Here, the absence of thermal energy means that the properties are instead driven by quantum fluctuations, leading to different scaling behaviors, and the degree to which such quantum phase transitions can be universally classified is still an open question [1]. In recent years, quantum criticality has become increasingly appreciated as underlying many of the novel phenomena arising in correlated materials. In particular, quantum fluctuations appear to play a key role in driving the breakdown of Landau Fermi liquid theory at finite temperatures near a QCP and the associated accumulated entropy allows the formation of unusual quantum phases of matter associated with different degrees of freedom, such as spin, orbital and charge [1,2].

**Figure 1.**
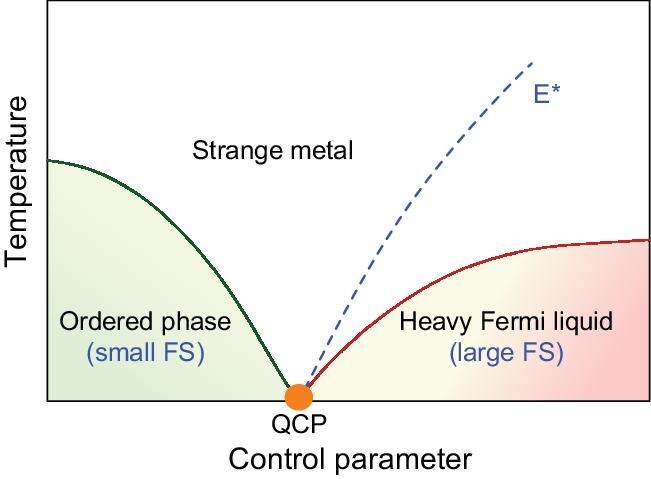
Schematic phase diagram for a Kondo-breakdown QCP [2]. Applying a non-thermal control parameter such as pressure or a magnetic field can eventually suppress an ordered phase, reaching a zero-temperature QCP. Beyond the QCP, a heavy-Fermi-liquid phase develops, in which the charge carriers incorporate the now delocalized magnetic moments, leading to an enhanced effective mass and an expansion of the Fermi surface (FS). These phases are separated by a fan-shaped region with strange-metal behavior. An energy scale E* is shown by the dashed line, signaling the delocalization of localized moments, and intersects the QCP; as a result, there is a ‘small FS’ in the ordered phase and a ‘large FS’ beyond the QCP.

Heavy-fermion metals are an established setting for antiferromagnetic quantum criticality, which occurs due to competition between the Ruderman–Kittel–Kasuya–Yosida (RKKY) interaction, which usually leads to antiferromagnetic ordering of the magnetic moments, and Kondo screening, which entangles local moments with conduction electrons to form a heavy Fermi liquid. If the delicate balance between these interactions can be adjusted by applying pressure, magnetic fields or chemical doping, the magnetic transition can often be suppressed to a QCP. While QCPs are most commonly associated with antiferromagnetism [1,2], in recent years, they have been discovered in a wider range of contexts, including in materials hosting ferromagnetic- and nematic-order parameters [3,4]. Such quantum phase transitions often exhibit anomalous scaling behaviors, most notably so-called strange-metal phases, in which the electrical resistivity scales linearly with the temperature. This has led to the suggestion that these correspond to an unconventional type of quantum criticality, namely a Kondo-breakdown QCP [2], at which the Kondo effect itself collapses and there is a transition from a magnet with a ‘small’ Fermi surface to a paramagnet with a large Fermi surface formed by itinerant heavy quasiparticles (Fig. 1). Consequently, the critical degrees of freedom at Kondo-breakdown QCPs are not only those of the magnetic-order parameter, but also those associated with the breakup of Kondo singlets and Fermi-surface reconstruction [2].

In a recent work published in *National Science Review*, Zhang and co-workers demonstrated a new possible scenario for quantum criticality in the disordered iron-based metal TiFe_0.7_Cu_0.4_Sb [5]. Random occupation of Fe and Cu sites creates a highly inhomogeneous landscape of local moments and RKKY couplings, stabilizing a cluster spin-glass ground state at low fields, in which there is slow, collective freezing of mesoscopic spin clusters rather than long-range order. By applying a small magnetic field, the authors drive this frozen cluster state into a coherent heavy-fermion liquid. Around a critical field of ∼0.1 T, the specific heat coefficient shows a broad logarithmic divergence and the magnetic Grüneisen parameter exhibits quantum-critical scaling, which are signatures of a QCP associated with the suppression of the spin-glass phase.

Theoretically, it has been suggested that, in the presence of strong disorder and frustration, a Kondo lattice can form glassy or liquid-like spin states and, in such materials, there may be a Kondo-breakdown-type quantum phase transition between a spin glass and a heavy-fermion metal. Evidence for such a scenario is found in Hall resistivity measurements of TiFe_0.7_Cu_0.4_Sb, which indicate a growing Fermi-surface volume with increasing magnetic field that could signal the gradual incorporation of localized electrons into itinerant quasiparticles. This motivates further studies to determine whether the Kondo-breakdown scenario shown in Fig. 1 is applicable to TiFe_0.7_Cu_0.4_Sb and, more generally, whether this is a common thread underlying quantum criticality in diverse systems beyond the paradigm of antiferromagnetism, including in ferromagnets, nematics and spin glasses [3–5].

These findings show TiFe_0.7_Cu_0.4_Sb to be a candidate for a new setting for Kondo-breakdown physics beyond clean, ordered magnets, in which the ordered phase is not antiferromagnetic or ferromagnetic order, but a cluster spin glass. Disorder and frustration prevent the formation of long-range magnetic order yet still allow Kondo screening to compete with RKKY interactions and can be tuned to criticality by using a tiny magnetic field. As a result, it provides a promising platform for exploring the interplay of glassy spin freezing, Fermi-surface reconstruction and strange-metal-like transport in the vicinity of quantum criticality.
